# Traditional Chinese Medicine and renal regeneration: experimental evidence and future perspectives

**DOI:** 10.1186/s13020-024-00935-9

**Published:** 2024-06-03

**Authors:** Denglu Zhang, Huihui Jiang, Xianzhen Yang, Sanxia Zheng, Yi Li, Shuai Liu, Xiangdong Xu

**Affiliations:** 1 Central Laboratory, Affiliated Hospital of Shandong University of Traditional Chinese Medicine, Jinan, China; 2 Shandong Key Laboratory of Dominant Diseases of Traditional Chinese Medicine, Affiliated Hospital of Shandong University of Traditional Chinese Medicine, Jinan, China; 3 Clinical Laboratory, Affiliated Hospital of Shandong University of Traditional Chinese Medicine, Jinan, China; 4 Urinary Surgery, Affiliated Hospital of Shandong University of Traditional Chinese Medicine, Jinan, China; 5 Pediatric Department, The Second Affiliated Hospital of Shandong University of Chinese Medicine, Jinan, China; 6 Department of Central Laboratory, Shandong Provincial Hospital Affiliated to Shandong First Medical University, Jinan, China; 7 Engineering Laboratory of Urinary Organ and Functional Reconstruction of Shandong Province, Shandong Provincial Hospital Affiliated to Shandong First Medical University, Jinan, China

**Keywords:** Acute kidney injury, Traditional Chinese Medicine, Stem cell, Regeneration, Macrophage, Peritubular capillary

## Abstract

Repair of acute kidney injury (AKI) is a typical example of renal regeneration. AKI is characterized by tubular cell death, peritubular capillary (PTC) thinning, and immune system activation. After renal tubule injury, resident renal progenitor cells, or renal tubule dedifferentiation, give rise to renal progenitor cells and repair the damaged renal tubule through proliferation and differentiation. Mesenchymal stem cells (MSCs) also play an important role in renal tubular repair. AKI leads to sparse PTC, affecting the supply of nutrients and oxygen and indirectly aggravating AKI. Therefore, repairing PTC is important for the prognosis of AKI. The activation of the immune system is conducive for the body to clear the necrotic cells and debris generated by AKI; however, if the immune activation is too strong or lengthy, it will cause damage to renal tubule cells or inhibit their repair. Macrophages have been shown to play an important role in the repair of kidney injury. Traditional Chinese medicine (TCM) has unique advantages in the treatment of AKI and a series of studies have been conducted on the topic in recent years. Herein, the role of TCM in promoting the repair of renal injury and its molecular mechanism is discussed from three perspectives: repair of renal tubular epithelial cells, repair of PTC, and regulation of macrophages to provide a reference for the treatment and mechanistic research of AKI.

## Introduction

Acute kidney injury (AKI) is a serious clinical syndrome with high morbidity and mortality, and no ideal clinical treatment [1]. AKI is primarily caused by proximal renal tubule injury, accompanied by microvascular injury and immune activation [2]. The kidneys are able to repair damage. Several studies have shown that kidney injury can activate resident kidney progenitor or tubular cells to dedifferentiate into kidney progenitor cells, promote their proliferation and differentiation, and participate in kidney injury repair [3, 4]. Additionally, studies have revealed that mesenchymal stem cells (MSCs) can promote the repair of kidney injury by regulating the innate immune balance [5]. In addition, MSCs can migrate to the kidneys, differentiate into renal parenchymal cells, and promote the regeneration of damaged kidney cells [6, 7]. AKI is typically accompanied by perirenal capillary damage, resulting in renal tubule hypoxia, which crucially affects the self-repair ability of the renal tubules and is an important factor in AKI-chronic kidney disease (CKD) transformation [8]. During AKI, various factors participate in the activation and recruitment of immune cells to the injured kidneys. These factors include damage-associated molecular patterns (DAMPs), hypoxia-inducible factors (HIFs), adhesion molecules, chemokines, and cytokines [9, 10] Immune cells of the innate and adaptive immune systems, such as neutrophils, dendritic cells (DCs), macrophages, and lymphocytes, are involved in the pathogenesis of kidney injury, and some of their subgroups are involved in the repair process [9, 10]. Traditional Chinese medicine (TCM) provides a theoretical basis for the treatment of AKI, vascular injury, and immune regulation. Herein, the research progress of TCM in promoting AKI repair is discussed from three perspectives: repair of renal tubular epithelial cells, repair of peritubular capillaries (PTC), and regulation of immune cells.

## Kidney injury repair and stem cells

**Fig. 1:**
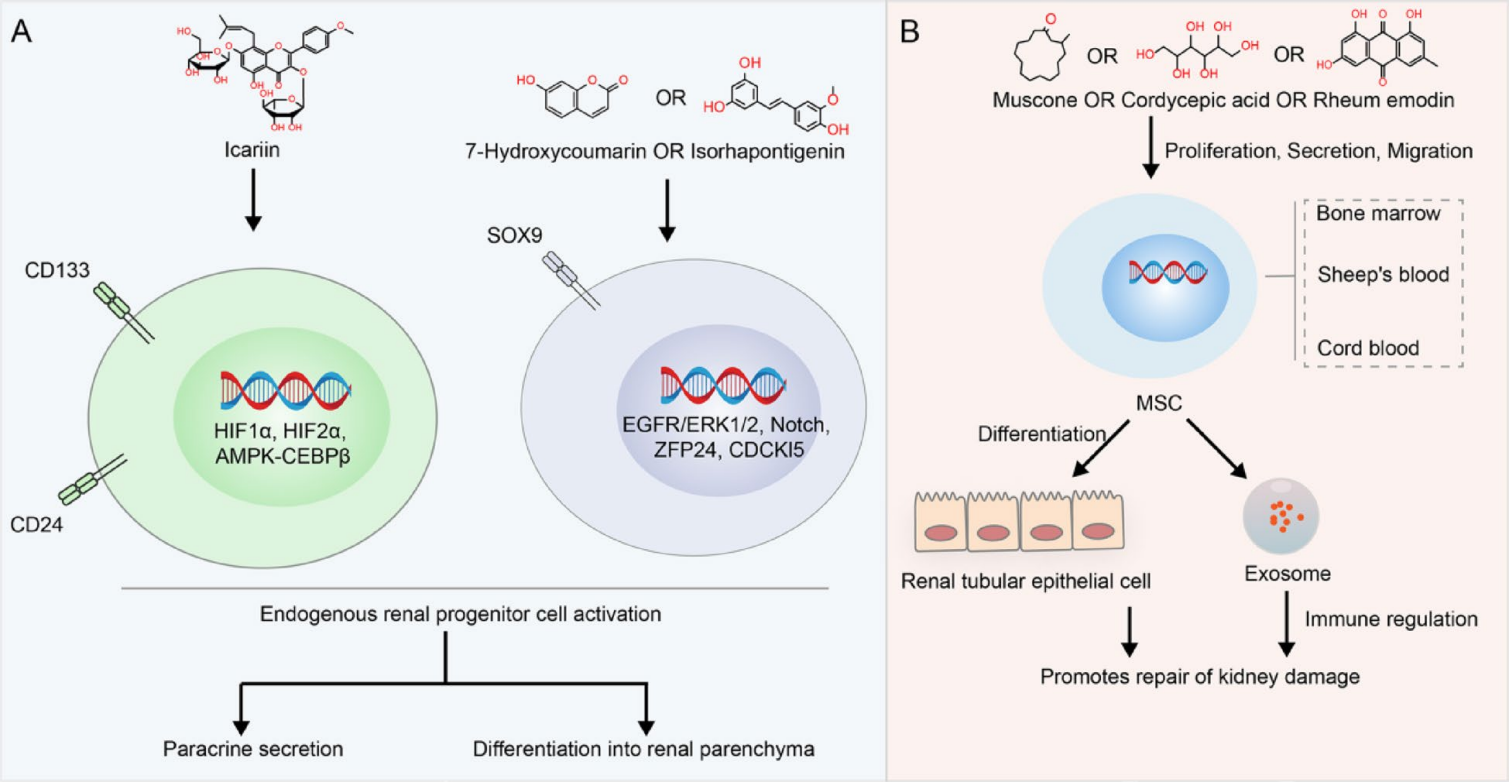
Mechanism of adult stem cells in acute renal injury repair and traditional Chinese medicine intervention

Stem cells are self-renewing cells with an infinite or immortal capacity to produce at least one type of highly differentiated daughter cell. Depending on their source, stem cells are divided into embryonic, adult, and induced pluripotent stem cells. Stem cells play a central role in the regenerative processes. Several adult organs contain stem cells. These cells are found in adult tissues and can differentiate into any cell type in the original organ. In contrast to embryonic stem cells, these cells are considered pluripotent instead of omnipotent. Progenitor cells are assumed to have a more limited differentiation capacity than stem cells and can differentiate into one or more cell types of the original tissue, however, can only replicate a limited number of times. In the kidneys, progenitor cells are typically at rest, and when activated by stimulation, they proliferate, eventually migrating to the site of injury and constructing novel renal tubules [3, 4]. I Currently, at least two types of kidney progenitor cells, CD133 + CD24 + and Sox9 + , are involved in kidney injury repair. Additionally, MSCs play an important role in AKI repair. Figure 1 describes how adult stem cells, including Renal progenitor cells and MSCs, are involved in the repair of AKI and how TCM is involved in the repair of AKI through the regulation of stem cells.

### Renal progenitor cells

#### Renal progenitor cells participate in renal injury repair

In the early 1900s, Jean Oliver described the process of renal tubular epithelial cell replication, replacing lost cells, and repairing injured epithelium [11]. Chang-Panesso et al. reported that after tubular death caused by AKI, tubular cells proliferate rapidly to restore the number of tubular cells, peaking 48 h after the injury. Pedigree tracing experiments have confirmed that these repair cells originate from the renal tubule instead of from circulating or interstitial progenitors [12]. Evidence suggests that the dedifferentiated epithelial cells that survive AKI have the same repair capacity [13–16]. In addition, a group of WNT-responsive or PAX2 positive or CD133-, CD24-, or SOX9-positive intracellular progenitor cells selectively proliferate and differentiate into renal tubular cells [17–19]. CD133 + CD24 + cells have been identified as the cellular mediators of proximal tubular repair in mice and humans, suggesting the possibility of an intratubular progenitor population [20]. Recently, several studies have shown that injury-induced Sox9 activation acts as a regenerative signal. Several studies have confirmed that Sox9 + renal tubular cells have progenitor cell properties, which increase rapidly after kidney injury and then participate in the repair of kidney injury [19, 21–23]. Briefly, following kidney injury, the renal tubules are initially damaged, prompting the proliferation of renal progenitor cells, which then contribute to the repair of damage.

Depending on the severity of the injury or compromised bodily functions, such as aging, kidney damage may not be repaired promptly, potentially leading to serious healthoutcomes. Several studies have shown that exogenous kidney progenitor cells participate in the repair of kidney injury and promote the recovery of kidney function [24, 25]. However, the clinical application of exogenous progenitor cells faces challenges due to immune rejection and safety problems, making it difficult to advance. The activation of endogenous kidney progenitor cells is a more promising option.

#### Potential targets for regulating renal progenitor cells

Low Sox9 expression has been observed in adult kidneys. However, when cellular stress or injury occurs, Sox9 undergoes transcriptional activation [21–23]. Kumar et al. discovered that in acutely injured mammalian kidneys, the activation and regulation of Sox9 are mediated by the EGFR/ERK1/2 signaling pathway and HIFA [4]. Ma et al. showed that kidney resection-induced kidney injury triggers the activation of Sox9 expression through the Notch signaling pathway, thereby facilitating the repair of the injury [22]. Similarly, Kim et al. observed that CDCKl5 exerts suppressive effects on Sox9, a transcriptional regulator associated with cell survival, through phosphorylation-dependent mechanisms in the context of renal injury [26]. Furthermore, Kim et al. identified an essential role for the ZFP24 protein in the activation of Sox9 during AKI [27]. These studies provide evidence of the effects of SOX9 expression or phosphorylation on renal injury repair.

A study conducted by Ohnishi et al. showed that HIF-1a activated the CD133 promoter in human embryonic kidney (HEK) 293 cells and the colon cancer cell line WiDr. One of two E-twenty-six (ETS) binding sites (EBSs) in the P5 region is required for its promoter activity induced by HIF-1a and HIF-2a. Immunoprecipitation experiments revealed that HIF-1a physically interacts with Elk1; however, HIF-2a does not interact with Elk1 or ETS1 [28]. Bussolati et al. reported that when CD133 + cells were cultured under hypoxiccondition in 1% oxygen, CD133 expression was upregulated after 24 h and was maintained for up to 72 h. Compared with CD133 + cells from the papillary region, CD133 + cells cultured under hypoxicconditions promote the rapid upregulation of HIF1, but not HIF2, which is constitutively expressed [29]. Maehara et al. demonstrated that metformin can inhibit the expression of CD133 in hepatocellular carcinoma cell lines through the AMPK-CEBP-β pathway [30].

#### TCM ameliorates kidney injury repair involved in the regulation of endogenous kidney progenitor cells

Wu et al. showed that 7-hydroxycoumarin (7-HC, also known as umbelliferone, commonly found in Chinese herbs such as Eucommiae Cortex, Prunellae Spica, Radix Angelicae Biseratae) inhibits necrosis and promotes the expression of Sox9 and proliferation of renal tubular epithelial cells, thus participating in kidney injury repair [31]. Experimental data have demonstrated that knockdown of Sox9 attenuates the 7-HC suppressive effects on KIM-1 and reverse the 7-HC stimulatory effects on cyclin D1 expression in HK-2 cells treated with cisplatin, indicating that the AKI protective mechanism stimulated by 7-HC may be mediated through Sox9 [31]. However, the mechanism by which 7-HC affects Sox9 expression remains unclear.

Zheng et al. used paraquat to induce AKI and isorhapontigenin as an intervention. This study showed that isorhapontigenin affected TOLLIP expression through the upregulation of Sox9, thereby reducing apoptosis and oxidative stress [32]. In NRK-52E cells, the overexpression of SOX9 demonstrated a mitigating effect on paraquat-induced apoptosis and oxidative stress. Conversely, SOX9 knockdown reduced the protective effects of isorhapontigenin. These findings suggest that SOX9 plays a crucial role in the therapeutic potential of isorhapontigenin for the treatment of paraquat-induced AKI [32].

Huang et al. used 5/6 nephrectomy to prepare a chronic renal failure model and demonstrated that icariin reduced creatinine and urea levels and promoted renal function recovery. Furthermore, icariin significantly increased the expression of CD133 and CD24 in renal tubular cells and promoted the proliferation of CD133 + /CD24 + renal progenitor cells [33].

All the aforementioned studies used AKI monomers to intervene and promote kidney injury repair by promoting the expression of kidney progenitor cell markers. The mechanism through which these monomers affect the expression of SOX9 or CD133\CD24 requires further investigation.

### MSCs promote renal injury repair

MSCs are multipotent cells derived from various sources such as the bone marrow, adipose tissue, and peripheral blood. MSCs can undergo in vitro expansion while preserving a relatively stable phenotype, enabling the cultivation of numerous cells suitable for clinical applications [34]. MSCs demonstrate the ability to relocate to areas of injury or inflammation and modulate both innate and adaptive immune reactions [35]. Furthermore, MSCs are recognized for their substantial involvement in tissue repair and regeneration, primarily attributed to the secretion of paracrine and endocrine signals with anti-inflammatory, anti-apoptotic, and pro-angiogenic properties [36].

The robust differentiation capacity of MSCs plays a notable role in facilitating tissue damage repair. Qian et al. demonstrated that MSCs derived from the bone marrow can mitigate AKI in rats by differentiating into cells resembling renal tubular epithelial cells [37]. In an AKI model, Li et al. observed that adipose MSCs transformed into renal tubular epithelial cells during the early stages of injury. This transformation assists in replacing necrotic cells, maintaining the integrity of the renal tubular structure, and contributing to tissue repair [38]. The effectiveness of MSC in AKI is primarily attributable to a paracrine mechanism [39]. Recent studies have also demonstrated that MSC can aid in the treatment of AKI through exosome secretion [40, 41] (Fig. 1B).

#### TCM can promote kidney injury repair by regulating MSCs

Musk has been used clinically as a natural TCM for thousands of years. Muscone, the chemical name for which is 3-methylcyclopentadecanone, is the main aromatic component of the natural Chinese medicinal musk. Musctone has anti-apoptotic and anti-oxidative stress properties and positively regulates the proliferation, secretion, and migration of bone marrow-derived MSC (BMSCs) to injured sites [42–44]. Liu et al. discovered that enhancing the bioactivity of BMSCs with muscone increased their therapeutic potential of BMSCs. These findings have important implications for the development of novel therapeutic approaches for the treatment of AKI [45].

Cordyceps is a traditional Chinese herbal medicinal plant genus. Such plants can be converted into a Bailing capsule through deep fermentation at low temperatures and their major component is cordycepic acid. Zhi-bo et al. showed that Bailing capsules combined with human amniotic MSCs can significantly improve adriamycin-induced nephrotic syndrome, and the improvement effect is significantly higher than that of human amniotic MSCs alone. Further studies have shown that Bailing capsules can promote the proliferation of human amniotic MSCs, thus achieving improved therapeutic effects [46].

Emodin, an anthraquinone derivative, is the main active component of rhubarb, and exhibits anti-inflammatory, antibacterial, immunomodulatory, and antioxidant properties. Studies have shown that emodin combined with BMSCs can improve ischemic reperfusion renal injury in rats more than BMSCs alone [47]. However, whether emodin affects BMSCs remains unclear.

The aforementioned studies focused on the treatment of renal injury using exogenous MSCs and Chinese medicine monomers. Whether Chinese medicine promotes kidney injury repair by regulating endogenous MSC has not yet been reported.

Some Chinese medicines and their active ingredients have been reported to regulate endogenous MSCs and have therapeutic effects against AKI. The relation of this treatment to the regulation of endogenous MSCremain uncler. For example, Jihong et al. reported that appropriate concentrations of *Astragalus* injection (0.05 g/mL), Astragalus IV injection (100 μmol/L), and Astragalus polysaccharide (1 mg/mL) can significantly promote the proliferation of BMSCs in rats [48]. These drugs have also received experimental support for AKI treatment [49–52]. Total saponins of Panax notoginseng can effectively improve myocardial remodeling after acute myocardial infarction, promote high expression of CD34 in the edge area of myocardial infarction, and promote the homing of CD34 + cells to the site of myocardial injury after acute myocardial infarction by the stem cell mobilizer G-CSF [53]. Notoginseng saponins can be used to treat or alleviate cisplatin-induced AKI [54]. Chinese herbs and active ingredients regulating MSC for the treatment of AKI are listed in Table 1.

**Table 1:** Active ingredients of Chinese medicine for treating AKI by regulating MSCs

TCM	Active ingredient	Treatment dose and administration methods	AKI models	Effect on MSC	References
Astragalus Membranaceus	Astragaloside IV	50–400 μM (cells); 2–10 mg/kg (rats, po.); 25–100 mg/kg (mice, po.)	IRI, LPS, cisplatin	Promote proliferation	[48–51]
	Astragalus polysaccharide	0.5–4.0 mg/mL (cells); 1–5 mg/kg (mice, po.)	LPS	Promote proliferation	[48, 52]
Epimedium	Icariin	30–60 mg/kg (mice, po.); 0.01–10 μM (cells)	Cisplatin, LPS	Promote proliferation	[55–57]
Panax ginseng	Ginsenoside	50 mg/kg (rats, i.p.); 1 μM (cells)	LPS	Ameliorating paracrine	[58, 59]
Carcuma longa	Curcumin	30–200 mg/kg (rats, po.); 50–200 mg/kg (mice, po.); 20 μM (cells)	IRI, LPS, Glycerol, Cisplatin	Ameliorating paracrine	[60–64]
Panax notoginseng	Panax Notoginseng Saponins	6.25 mg/mL((cells); 150 mg/kg (rats, po.)	Cisplatin	Facilitated homing	[54, 65]
Ligusticum wallichii	Ligustrazine	10–60 mg/kg (mice, i.v.); 10–200 μM (cells)	LPS, IRI	Facilitated homing	[66–68]
Polygonum cuspidatum	Resveratrol	50 or 200 μM (cells)	LPS	Inhibit senescence	[69, 70]
Dendrobe	Naringenin	10 and 20 mg/kg (rats, po.); 25 μM (cells)	LPS	Inhibit senescence	[71, 72]

*po.* oral administration, *i.v.* intravenous injection, *i.p.* intraperitoneal injection

## PTC injury and repair

### PTC and AKI

Sparse PTC is a major feature of the kidney after AKI and a risk factor for AKI-CKD transformation [73]. AKI caused by renal ischemia–reperfusion injury can lead to the cytoskeletal rearrangement of endothelial cells and damage their tight connections. After injury, capsase-3 is activated in the endothelial cells, inducing apoptosis, and leading to vascular thinning [74]. After the onset of AKI, pericytes release anti-angiogenic molecules such as ADAM metallopeptidase with thrombospondin type 1 motif 1(ADAMTS-1), which, along with inhibiting the downregulation of tissue metalloproteinase-3, threaten vascular stability and leads to capillary detachment [75]. In addition to ischemic AKI, sparse PTC has been observed in toxic and obstructed AKI/CKD models [76, 77]. The signaling pathways that trigger PTC sparring are activated early after AKI and are notable obstacles in effective renal repair and recovery [75].

### Promoting PTC regeneration can promote AKI repair

**Fig. 2:**
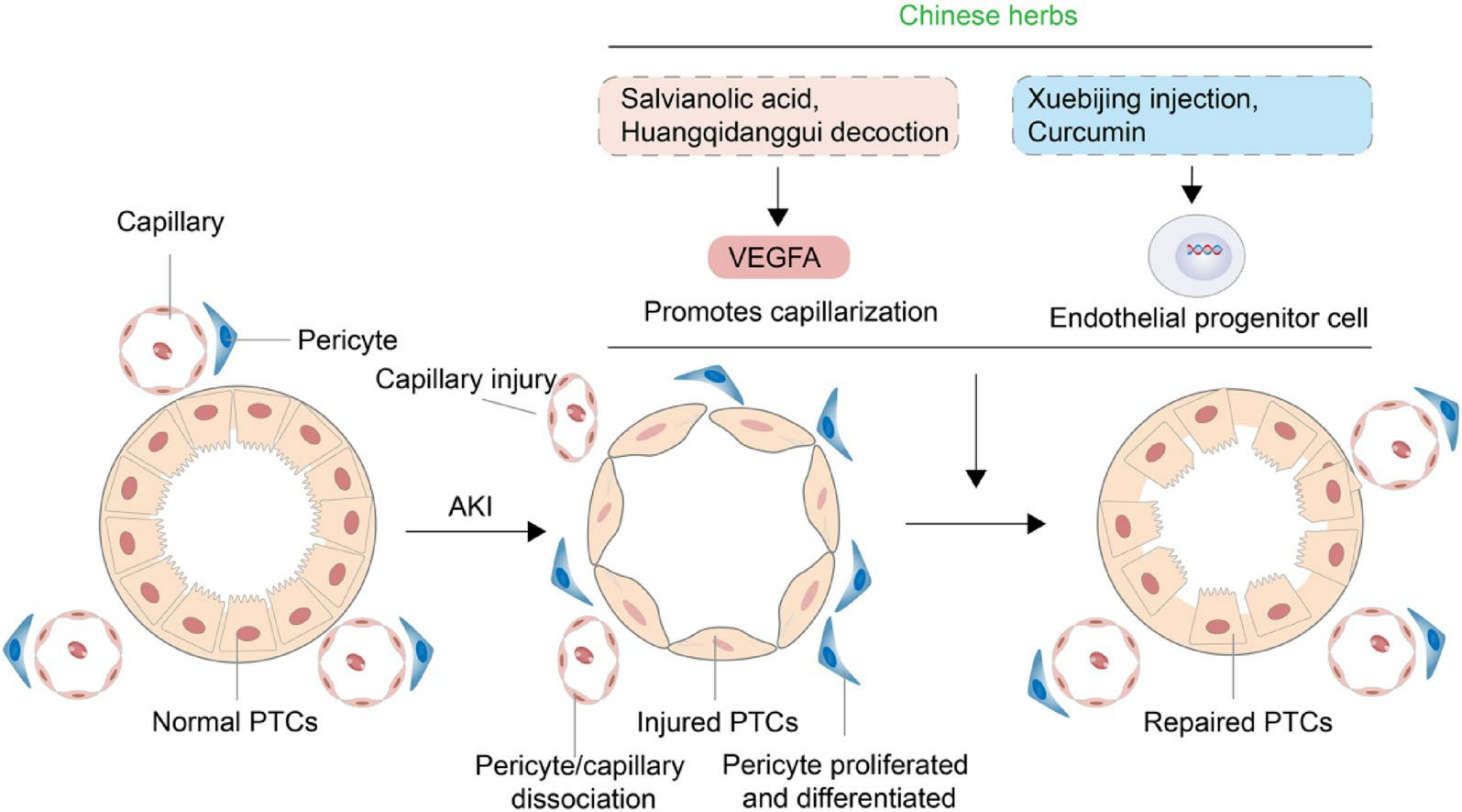
Traditional Chinese medicine is involved in the repair mechanism of AKI by intervening in vascular regeneration

Previous studies have shown that renal tubule cells promote PTC proliferation by secreting extracellular vesicles containing VEGF-A, whereas the addition of exogenous extracellular vesicles containing VEGF-A can promote PTC proliferation and kidney injury repair in ischemic AKI models [78]. BMSC transplantation can treat ischemic AKI, and its primary mechanism is to promote the repair of kidney injury by repairing PTC and increasing the PTC density [79]. Additionally, the activation of the angiopoietin-Tie2 signal through the regulation of endothelium-specific signaling pathways plays a protective effect in kidney injury caused by ischemia [80]. TCM played an important role in the repair of PTC for the treatment of AKI, which was reviewed as followsas shown in Fig. 2.

### TCM promote the repair of kidney injury by promoting capillary regeneration through various mechanisms

#### VEGF and other pro-angiogenic factors

Zhang et al. showed that salvianolic acid A alleviates AKI caused by ischemia and reperfusion. Salvianolic acid A maintaines PTC density by promoting VEGF-A expression and alleviating kidney damage caused by hypoxia [81]. Song et al. reported that an Astragalus danggui decoction improved renal function in 5/6 nephrectomized rats. Further studies have reported that Astragalus danggui decoction promotes the regeneration of PTC and glomerular capillaries through the upregulation of VEGF, thus improving renal function [82].

#### Endothelial progenitor cells (EPCs)

The major components of Xuebijing injection are red peony root, Chuanxiongxiong, Salvia miltiorrhiza, safflower, and angelica, which can antagonize endotoxins, improve microcirculation, protect endothelial function, and alleviate AKI caused by endotoxins in mice [83]. Jian et al. reported that Xuebijing injection promoted the proliferation, migration, and tubule formation ability of renal microvascular endothelial cells and the expression of VEGF and fibroblast growth factor 2 (FGF2), and enhanced the repair effect of EPCs on the damage of renal microvascular endothelial cells induced by Lipopolysaccharide (LPS) [84].

Curcumin is a polyphenolic compound extracted from the rhizome of the Curcuma genus that has a wide range of pharmacologicalprosperties, including anti-inflammatory, antioxidant, and antiviral properties [85]. Qi et al. investigated the effects of curcumin pre-treatment on vascular endothelial repair and EPCs homing in the renal tissue of rats with ischemia–reperfusion-induced AKI. This study confirmed that curcumin pretreatment could promote the homing of EPCs to the kidney to repair damaged endothelial cells around the capillaries, thereby reducing renal tubule injury and alleviating ischemia–reperfusion-induced AKI [86].

Numerous TCMs or active ingredients, including Astragalus and Salvia miltiorrhiza [87, 88], promote angiogenesis, and are widely used to treat kidney injury [49–51] (Table 2). The topic, whether these drugs promote kidney injury repair by promoting angiogenesis requires further investigation.

**Table 2:** Chinese medicine ingredients that can treat AKI by promoting angiogenesis

TCM	Active ingredient	Treatment dose and administration methods	AKI models	Angiogenesis mechanism	References
Astragalus Membranaceus	Astragaloside IV	0.1–10 mg/kg (rats, po.); 25–100 mg/kg (mice, po.)	IRI, LPS, Cisplatin	VEGF and FGF2	[49–50, 87]
	Astragalus polysaccharide	1–5 mg/kg (mice, po.); 10–100 μg/mL (rats, po.)	LPS	AKT/eNOS	[52, 88]
Salvia miltiorrhiza Bunge	Salvianolic acid A	2.5–24 mg/kg (rats, i.v.); 100–120 mg/kg (rats, po.)	IRI	VEGF	[81, 89]
	Salvianolic acid B	50–200 mg/kg (mice, po.); 50 μg/mL (zebrafish)	IRI	VEGF	[90, 91]
Rhodiola Crenulata	Salidroside	50–150 mg/kg (rats, i.v.); 100 nM (cells)	LPS	HIF-1a/VEGF	[92, 93]
Pueraria	Puerarin	25–100 mg/kg (rats, i.v.); 100 μM (cells)	Cisplatin	VEGF	[94, 95]
Panax ginseng	Ginsenoside	50 mg/kg (rats, i.p.); 10–40 mg/kg (mice, i.p.)	LPS	VEGF	[58, 96]
Epimedium	Icariin	30–60 mg/kg (mice, po.)	LPS, Cisplatin	VEGF, Tie2	[55, 97]
Panax notoginseng	Notoginsenoside	80 or 100 μM (zebrafish); 20 or 40 mg/kg (rats, i.p.)	Acetaminophen	Ang2/Tie2	[98, 99]

*po.* oral administration, *i.v.* intravenous injection, *i.p.* intraperitoneal injection

## Macrophages and kidney injury repair

**Fig. 3:**
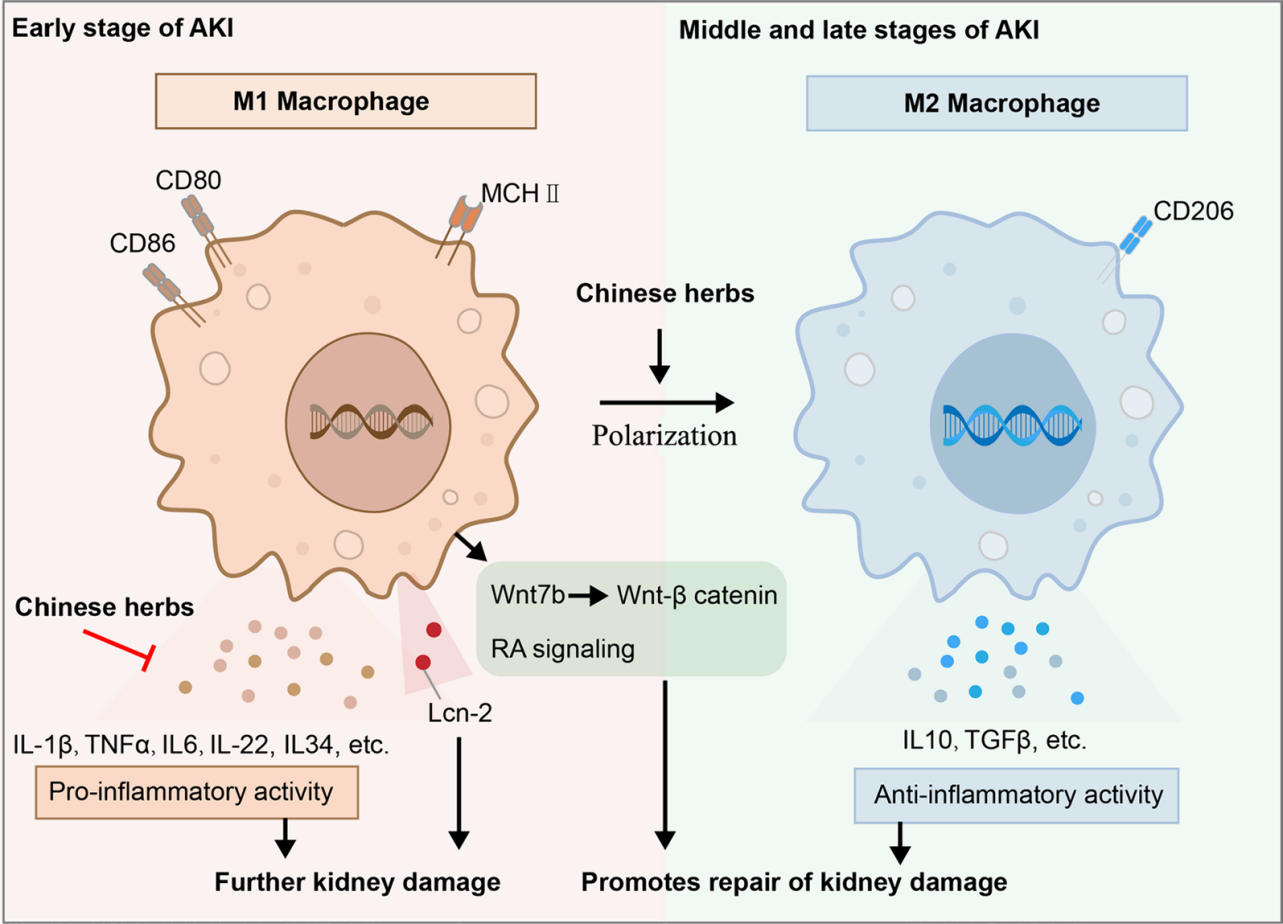
Mechanism of macrophages involved in the repair of AKI and the intervention of TCM

Macrophages are crucial cellular components for the restoration of renal function, exhibiting important functions beyond their well-established proinflammatory properties. Macrophages play a pivotal role in wound healing and facilitate regeneration by bridging the gap between the initial inflammatory response and the subsequent phases of tissue regeneration and repair. However, the prolonged and persistent presence of macrophages in tissues can potentially prolong the damage phase, ultimately leading to failure of tubular repair. This failure contributes to maladaptive kidney repair and plays a role in the transition from AKI to CKD [100, 101]. Figure 3 describes the mechanism of macrophages participating in the repair of AKI, and how Chinese medicine promote the repair of kidney injury through the intervention of macrophages.

### Mechanisms of macrophages involved in renal injury repair

#### Retinoid acid (RA) signaling

The RA signaling pathway plays a key role in kidney development [102], and its reactivation has an important function in the repair of kidney injury [103]. Macrophage-driven RA signaling within proximal tubular epithelial cells (PTECs) has been hypothesized to contribute to the repair of these cells. This hypothesis was supported by a study using RA signaling reporter mice, specifically RARE-hsp68-LacZ mice, which express the β-galactosidase gene under the control of the RA-responsive element. These mice showed activation of the reporter in injured PTECs within 12–24 h after injury, with persistence up to 72 h and a return to baseline levels by day 7. Further investigation revealed that locally synthesized RA inhibits proinflammatory macrophages, leading to a reduction in macrophage-dependent injury following AKI. In addition, the activation of RA signaling in the injured tubular epithelium promotes alternatively activated M2 spectrum macrophages [104].

#### Secreting cytokines such as IL-22

Research has shown that interstitial mononuclear cells, specifically DCs and macrophages, are the primary contributors to the secretion of IL-22. Conversely, the expression of the IL-22 receptor is exclusively shown in tubular epithelial cells in kidneys [105, 106]. When IL-22-producing cells are depleted during the healing phase, they negatively affect epithelial recovery. However, in a recent study, this impairment was completely reversed when mice were reconstituted with IL-22 [107]. Another study demonstrated that IL-22 protected against ischemic AKI. Transgenic animals with IL-22 exhibited significantly higher survival rates, whereas knockout mice had a heightened mortality rate compared to wild-type (WT) mice [108].

#### Lipocalin-2 (Lcn-2) secretion

Macrophages promote the regeneration of renal tubular epithelial cells after kidney injury in mice by secreting Lcn-2 [109]. Brown Norway rats exhibited endogenous resistance to ischemia-induced kidney damage.Conversely, Sprague–Dawley (SD) rats showe a higher susceptibility to ischemic injury. Tolerant macrophages of Brown Norway rats express significantly higher levels of Lcn-2. In vivo studies have shown that after Lcn-2 knockdown in macrophages, renal tubular epithelial cell apoptosis and kidney injury in Brown Norway rats increase significantly and repair markers have decrease significantly, whereas lipocalin-2-overexpression cells have significantly decreased susceptibility in SD rats [110].

#### Wnt-β catenin signaling

The Wnt signaling pathway plays a key role in kidney development, and its reactivation plays an important role in tissue damage repair [17]. Macrophages within the kidney serve as both sources and recipients of WNT ligands [111]. Following injury, macrophages release WNT7B, which stimulates the repair and regeneration of interstitial and epithelial cells. In healthy adult kidneys, the canonical Wnt-β catenin pathway activity is limited to the papilla, whereas injury-induced pathway activation occurs in the cortical and medullary regions by day 5 post-injury [111].

#### Macrophage polarization

Macrophages exhibit notable plasticity in their ability to adapt to various environmental conditions and their function in the context of damage or repair is contingent on their specific phenotypes. M1 macrophages have been hypothesized to function as inflammatory cells that initiate kidney damage by releasing pro-inflammatory cytokines such as IL-6, TNFα, and IL-1β, whereas M2 macrophages play a crucial role in facilitating the restoration of kidney tissue[112, 113].

The M1/M2 macrophage ratio changes during AKI occurrence and development. In the immediate aftermath of injury, pro-inflammatory (classically activated M1) macrophages are recruited. These macrophages phagocytose cell debris, secrete cytotoxic molecules such as nitric oxide synthase (NOS) and reactive oxygen species (ROS), and induce mitochondrial damage and apoptosis [114]. Infiltrating cells release anti-inflammatory cytokines, including IL10 [115], IL4, and IL13 [116], to reverse the inflammatory environment and promote repair. These infiltrating cells include repair-promoting macrophages (type M2), CD4 + \CD8 + T cells, and regulatory T (Treg) cells. M2 macrophages produce arginase, an enzyme necessary to produce ornithine and polyamines, which are building blocks of the extracellular matrix architecture [117]. Macrophages may also participate in the repair of kidney injury by promoting angiogenesis and anti-inflammation [117].

#### TCM plays a role in the repair of AKI by regulating macrophages

In clinical studies, renal macrophages increase when AKI and the M2 macrophage marker CD163 is detected on the surface of 75% of macrophages in the early repair stage [118, 119]. Danhong injection alleviates AKI caused by ischemic reperfusion, partly by reducing macrophage infiltration [120]. Chen et al. showed that resveratrol can alleviate LPS-induced AKI, mainly through inhibiting the release of inflammatory factors by macrophages and the activation of TLR4 [121]. Yan et al. determined that rhabdosin alleviates AKI caused by ischemia/reperfusion. Its mechanism primarily involves the inhibition of the inflammatory response of macrophages by inhibiting the AKT signaling pathway [122]. Weijia et al. reported that astragaloside can reduce the M1 polarization of macrophages, levels of inflammatory factors IL-6 and TNF-α, and macrophage activity, thus playing a role in slowing down the kidney damage of aristolochic acid. This mechanism may be related to the partial inhibition of p38 MAPK signaling activity [123].

In the aforementioned studies, TCM played an anti-inflammatory role by reducing injury and promoting injury repair.

#### Regulating the balance of M1/M2 macrophage polarization may be an effective therapeutic target in AKI

Several studies have shown that regulating of macrophage M2 polarization can promote the repair of kidney injury after AKI. As mentioned, local synthesis of RA inhibits the activation of proinflammatory macrophages, leading to a decrease in macrophage-mediated damage following AKI. Additionally, RA signaling is stimulated in the injured tubular epithelium, thereby facilitating the development of alternatively activated M2 spectrum macrophages [104]. The efficacy of EPO in mitigating kidney injury has been demonstrated through its ability to decrease macrophage recruitment and facilitate the transition from M1 to M2 macrophages in vivo [124]. Furthermore, in vitro studies revealed that EPO directly inhibits the proinflammatory response of M1 macrophages and enhances the expression of M2 markers [124].

Several studies have shown that berberine promotes the transformation of macrophages into M2 macrophages. Yang et al. showed that in chronic atrophic gastritis induced by *Helicobacter pylori*, berberine regulates macrophage polarization through the IL-4-STAT6 signaling pathway, inhibits the M1 type, and promotes M2 type transformation [125]. Similar results were observed in a mouse model of ulcerative colitis [126]. Lin et al. reported that berberine reduced adipose tissue inflammation in mice fed a high-fat diet. This promotes the transformation of macrophages into the M2 type [127]. Gao et al. reported that curcumin promotes the secretion of IL4 and or IL13 by macrophages and the polarization of M2 macrophages in experimental autoimmune myocarditis models [128]. In addition, the active ingredients in Chinese medicines, such as astragaloside, diosgenin, ginsenoside Rg1, Lupeol, and Platycodin D, promote the polarization of M2 macrophages [129–133]. However, whether berberine [134], curcumin [85], astragaloside [49–51], diosgenin [135], and ginsenoside Rg1 [58] used in the treatment of AKI (Table 3) are related to the promotion of polarization of M2 macrophages requires further study.

**Table 3:** Chinese medicinal ingredients used to treat AKI by promote the polarization of macrophages

TCM	Active ingredient	Treatment dose and administration methods	AKI models	Effect on macrophages	References
Astragalus Membranaceus	Astragaloside IV	2–40 mg/kg (rats, po.); 25–100 mg/kg (mice, po.)	IRI, LPS, Cisplatin	M2 polarization	[49–50, 129]
Dioscorea nipponica Makino	Dioscin	0.625–2.5 μM (cells); 40–160 mg/kg (mice, po.); 60 mg/kg (rats, po.)	Cisplatin	M2 polarization	[128, 135]
Panax ginseng	Ginsenoside Rg1	50 mg/kg (rats, i.p.); 200 mg/kg (mice, po.)	LPS	M2 polarization	[58, 131]
Coptis chinensis	Berberine	14–28 mg/kg (rats, po.); 20–100 mg/kg (mice, po.); 5–10 mg/kg (mice, i.p.)	Cisplatin	M2 polarization	[125–127, 134]
Carcuma longa	Curcumin	50 mg/kg (rats, po.); 200 mg/kg rats, i.p.)	IRI	M2 polarization	[86, 128]

*po.* oral administration, *i.v.* intravenous injection, *i.p.* intraperitoneal injection

## Conclusions and perspectives

The use of drugs and therapies to mobilize the body’s own ability to eliminate disease and restore health is a primary feature of TCM. Several human organs and tissues exhibit varying degrees of regenerative potential, which can be activated under certain conditions and play a role in treating diseases. In the past, the kidneys were assumed to have no regenerative capacity. However, with progress in science and technology, increasing evidence has shown that the kidney has a certain regenerative ability, especially in the repair process of AKI [3, 4]. TCM scholars assume that Jing is fundamental for human development, regeneration, repair and maintenance of life. Jing has a similar role and status to stem cells in regenerative medicine. When the kidney injury is relatively serious, an imbalance occurs between the kidney injury and the regenerative ability, which requires drug intervention. An increasing number of studies have shown that TCM plays an important role in the treatment of AKI [31–33]. However, most studies have focused on the protective effects of Chinese herbs or monomers against kidney damage, and the mechanisms typically include the inhibition of apoptosis as well as anti-inflammatory and antioxidant effects. Such research predominantly highlights the perspectives of TCM that involves eliminating disease-causing factors, while overlooking its crucial role in strengthening the body’s natural defenses, thus failing to capture the holistic essence of TCM..

In this study, we discussed the mechanisms of TCM in renal tubule regeneration, microvascular regeneration, and immune cell regulation in renal injury, staring from the mechanism of renal regeneration. Renal tubule injury is the primary feature of AKI and repairing this damage is central to the treatment of AKI. Promoting the proliferation and differentiation of renal progenitor cells and activating MSCs are key to promoting renal tubule regeneration and repair. Recent studies have found that 7-HC and isoflavin can promote the regeneration and repair of renal tubules [31, 32]. Other drugs that can promote the Sox9\CD133\WNT signaling pathway or activate MSCs (Table 1) may also play a role in the treatment of AKI; however, further investigation is required.

Micro vessel thinning is another characteristic of AKI and is closely related to prognosis. Recent studies have confirmed that the Chinese herbal compounds *Astragalus* danggui decoction, Xuebijing, traditional Chinese monomer salvianolic acid A, and curcumin can promote kidney injury repair by promoting vascular regeneration[81–83, 86].Other studies have reported that several microvascular regeneration drugs (Table 2) may also be effective in the treatment of AKI; therefore, further studies are required.

Macrophages play an indispensable role in the repair of renal injury. Studies on the influence of TCM on the prognosis of AKI by macrophage intervention have focused on the inhibition of macrophage infiltration or the reduction of macrophages. The regulation of macrophage M2 polarization promotes the repair of kidney injury after AKI [104, 122]. Currently, no reports are available, on the use of TCM or its active ingredients to interfere with macrophage M2 polarization and promote AKI repair, which may thus be the required direction of future research.

In conclusion, starting from the mobilization of the regenerative potential of the kidneys, this study discusses the research progress on TCM in the regulation the damage repair of renal tubular epithelial cells, damage repair of PTC, and immunity by TCM and presents insights to provide research foundations in this field.

## Data Availability

No data was used for the research described in the article.
